# Ergosterol Alleviates Kidney Injury in Streptozotocin-Induced Diabetic Mice

**DOI:** 10.1155/2015/691594

**Published:** 2015-11-17

**Authors:** Li Ang, Liu Yuguang, Wang Liying, Zhang Shuying, Xu Liting, Wang Shumin

**Affiliations:** Changchun University of Traditional Chinese Medicine, Jilin, Changchun 130117, China

## Abstract

Ergosterol (ERG) has been widely used in the development of novel drugs due to its unique physiological function. However, little is known about the protective effects of ERG on diabetes. Hence, the current study was designed to evaluate the positive role of ergosterol on streptozotocin- (STZ-) induced diabetes in mice. Oral glucose tolerance test (OGTT) was carried out to assess blood glucose level. Biochemical parameters such as uric acid, creatinine, serum insulin, triglycerides (TG), and total cholesterol (TC) were also measured. Pathological condition of kidney was examined by hematoxylin-eosin (H&E) staining. The expressions of PI3K, p-PI3K, Akt, p-Akt, NF-*κ*Bp65, p-NF-*κ*Bp65, I*κ*B*α*, and p-I*κ*B*α* were analyzed by western blot. ERG significantly reduced the concentrations of blood glucose, uric acid, creatinine, TG, and TC. Serum insulin was elevated with ERG treatment. In addition, renal pathologic changes of diabetes mice were also alleviated by ERG. Obtained data revealed that ERG restored the levels of PI3K/Akt/NF-*κ*B signaling-related proteins in comparison with diabetes mice. Above all, it could be assumed that ERG might play a positive role in regulating STZ-induced diabetes through suppressing PI3K/Akt/NF-*κ*B pathway.

## 1. Introduction

Ergosterol, extracted from oat ergot fungus in 1889 firstly, is a principal component of the fungal plasma membrane and has been extensively researched for decades [1, 2]. Besides, the biosynthetic pathway of ergosterol and genetic engineering to obtain high yield strains of ergosterol has been also rapidly developed [3], which largely contributes to the further exploration. It not only serves as an important pharmaceutical and chemical raw material but also functions as a key raw material in the production of steroid drugs since it can be converted to vitamin D2 when subjected to ultraviolet radiation [4, 5]. However, there are rarely reports focusing on the pharmacological effects of ergosterol. It is well known that appropriate supplementation of vitamin D, especially vitamin D2 [6], can increase insulin sensitivity, lower blood glucose, and reduce the cardiovascular complications of diabetes [7, 8]. Taking the partially similar functions with vitamin D into consideration, we thought it was worth studying whether ergosterol could improve STZ-induced diabetes.

Diabetes mellitus is a serious and complex metabolic disease which is called “The Silent Killer” due to the large number of patients and many chronic complications [9]. It is caused by decreased tissue response to the insulin and/or impaired insulin deficiency and is characterized by elevated blood glucose [10]. There are 387 million people living with diabetes nowadays and the number is expected to reach 592 million by 2035 according to the International Diabetes Federation [11]. Diabetes patients fail to fully utilize glucose so that it accumulates in the blood leading to high blood glucose [12]. Consequently, kidney is involved and metabolites in urine, such as uric acid and creatinine, are also upregulated. Secondary to the absolute or relative lack of insulin, disturbances in carbohydrate, protein, and fat metabolism occurred [13], which might be reflected by high levels of TG and TC.

Glucose is used in target tissues mainly by two pathways, phosphatidylinositol-3-kinase (PI3-kinase) and 5′-AMP-activated kinase (AMPK) [14]. Glucose homeostasis is broken by the absence of the AMPK and PI3K/Akt pathways in insulin-sensitive tissues, leading to the accumulation of glucose in the blood. It has also been documented that PI3K/Akt is one of the major signaling pathways, which is recognized as a major mechanism associated with the development of insulin resistance [15, 16]. Therefore, the investigation was conducted to evaluate the effect of ergosterol on STZ-induced diabetes mice through the PI3K/Akt signaling pathway.

## 2. Materials and Methods

### 2.1. Main Reagents and Kits

Ergosterol (ERG, purity 98%) was purchased from National Institutes for Food and Drug Control (Beijing, China). Allopurinol (ALL) tablets were acquired from Simcare Drug Store (Nanjing, China). Streptozotocin (STZ) was provided by Sigma (St Louis, MO, USA). Insulin enzyme-linked immunosorbent assay (ELISA) kits were supplied by Nanjing KeyGEN Biotech. Co., Ltd. (Nanjing, China). Glucose, triglycerides (TG), total cholesterol (TC), uric acid, and creatinine commercial kits were purchased from Jiancheng Bioengineering Institute (Nanjing, China). Primary antibodies against PI3K (p85 subunit), p-PI3K (p85 subunit), Akt, p-Akt, NF-*κ*Bp65, p-NF-*κ*Bp65, IкB*α*, and p-IкB*α* were produced by Cell Signaling Technology Inc. (Beverly, MA, USA).

All other chemicals and reagents used for study were of analytical grade and were purchased from approved organizations.

### 2.2. Animals

40 male mice, (8 weeks) weighing 20–22 g, obtained from Comparative Medicine Centre of Yangzhou University, were maintained in a temperature and humidity controlled animal facility with a set of 12 h light-dark cycle. Mice were provided with water and food pellets* ad libitum*. All mice were required to acclimatize to the laboratory environment for 7 days before the start of the experiment.

### 2.3. Experimental Protocol

Mice were divided into the five groups randomly as follows (*n* = 8 per group): control group, STZ group, STZ + ALL (5 mg/kg, orally) group, STZ + ERG (50 mg/kg, orally), and STZ + ERG (100 mg/kg, orally) group. A single dose of 135 mg/kg of STZ was injected intraperitoneally in citrate buffer at pH 4.2−4.5 prepared at the time of use. ALL and ERG were treated for consecutive 28 days. ERG (50, 100 mg/kg) and ALL (5 mg/kg) were intragastrically administrated by gavage.

### 2.4. Sample Preparation

After the treatment with allopurinol or ergosterol for 28 days, urine was collected in metabolic cages for 24 h on day 29. Mice were sacrificed on day 30. Blood samples were collected from the orbit and centrifuged at 4500 rpm for 15 min. Kidney was harvested subsequently and set aside at −80°C for histological observation and protein quantification.

### 2.5. Oral Glucose Tolerance Test (OGTT)

Mice in groups were orally administered with glucose (1.5 g/kg body weight) on day 28. The blood glucose level was monitored via the blood obtained from the tail vein using glucose commercial kits at 0 min (prior to glucose administration), 30 min, 60 min, 90 min, and 120 min, respectively.

### 2.6. Insulin Assay

The serum insulin level of each blood sample was measured by an enzyme-linked immunosorbent assay using a commercial kit according to the manufacturer's instruction.

### 2.7. Biochemical Analysis

Uric acid and creatinine, serum triglycerides (TG), and total cholesterol (TC) were detected by commercial kits on the basis of the manufacturer's instruction.

### 2.8. Histological Analysis of Kidney

Renal tissues were fixed in 10% (V/V) neutral buffered formalin for 24 h, embedded in paraffin wax, cut into 4 *μ*m thicknesses, deparaffinized in xylene, and processed with graded ethanol series. Sections were stained with stained with Hematoxylin and Eosin (H&E) and observed by light microscopy (Nikon, Tokyo, Japan) at 200x magnification.

### 2.9. Western Blot

Briefly, renal tissues (100 mg) were minced and homogenized in ice-cold 1 mL RIPA buffer, followed by centrifugation at 12000 rpm for 5 min at 4°C. The supernatant was collected to a new clean centrifuge tube and Bicinchoninic acid (BCA) protein assay kit (Beyotime, Nanjing, China) was used for quantification. Equal amounts of 30 *μ*g protein were subjected to a 10% SDS-polyacrylamide gel electrophoresis and transferred onto PVDF membranes. The target blots were incubated in blocking solution containing 5% skim dried milk. Subsequently, the membrane was exposed to the appropriate concentration of specific antibody overnight at 4°C. The incubation with horseradish peroxidase-conjugated second antibody was carried out at room temperature for 1 h after washing with TBST for three times. The antibody-reactive sheets were visualized by an enhanced chemiluminescence (ECL) advanced kit and a gel imaging system (Tanon Science & Technology Co., Ltd., China).

### 2.10. Statistical Analysis

All data were presented as means ± standard deviations (SDs). One-way analysis of variance (ANOVA) followed by Tukey's multiple comparison test was conducted to assess differences between groups. The values were considered statistically significantly different at *P* < 0.05. Calculations were made using GraphPad Prism.

## 3. Results

### 3.1. Change of the Blood Glucose and Serum Insulin Levels

**Figure 1:**
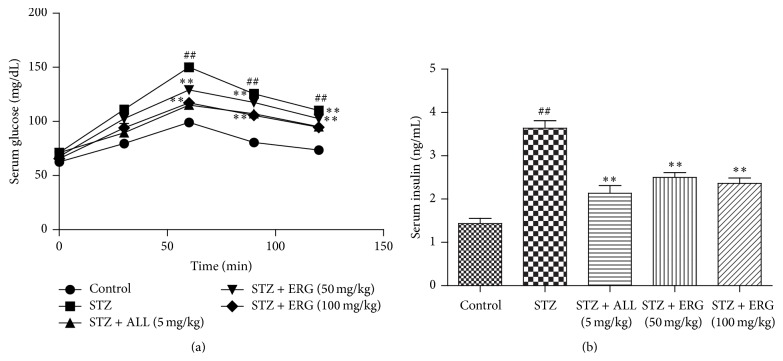
Effects of ERG on the blood glucose and serum insulin levels. (a) OGTT; (b) serum insulin levels. Mice were intraperitoneally injected with 135 mg/kg of STZ. ALL (5 mg/kg) and ERG (50, 100 mg/kg) were intragastrically treated for consecutive 28 days. Values are expressed as means ± SDs. Compared with control: ^##^ *P* < 0.01; compared with model: ^*∗∗*^ *P* < 0.01.

As shown in Figure 1, STZ stimulation led to the upregulation of blood glucose (*P* < 0.01) (Figure 1(a)) and downregulation of serum insulin content (*P* < 0.01) (Figure 1(b)). However, ERG (50, 100 mg/kg) treatment significantly recovered the abnormal levels and showed the potential to maintain blood glucose balance (*P* < 0.01). Serum glucose levels presented an apparent peak locating at 90 min. There was little difference at 90 min in the serum glucose between ALL (5 mg/kg) and ERG (50, 100 mg/kg), with ERG (100 mg/kg) showing higher decrease than ERG (50 mg/kg). Meantime, the effect of ALL treatment on the serum insulin level was slightly more pronounced than those of ERG (50, 100 mg/kg) treatments.

### 3.2. TG and TC Levels in Groups

**Figure 2:**
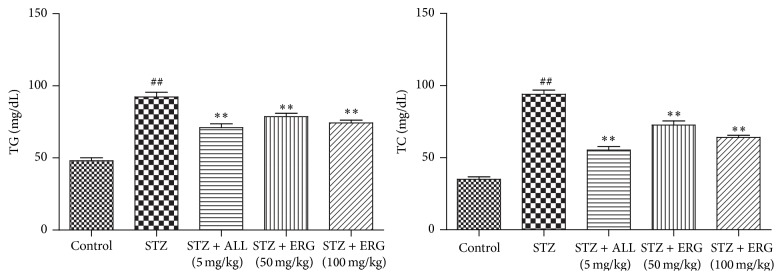
Effects of ERG on TG and TC levels. Mice were intraperitoneally injected with 135 mg/kg of STZ. ALL (5 mg/kg) and ERG (50, 100 mg/kg) were intragastrically treated for consecutive 28 days. Values are expressed as means ± SDs. Compared with control: ^##^ *P* < 0.01; compared with model: ^*∗∗*^ *P* < 0.01.

Serum TG (*P* < 0.01) and TC (*P* < 0.01) levels in STZ-treated group were significantly elevated compared with those in control group, while ERG (50, 100 mg/kg) administration markedly reduced the abnormal high expressions of them in diabetes mice (*P* < 0.01), which were little less efficient than that of ALL administration (*P* < 0.01) (Figure 2).

### 3.3. Uric Acid and Creatinine Concentrations in Diabetes and Control Mice

**Figure 3:**
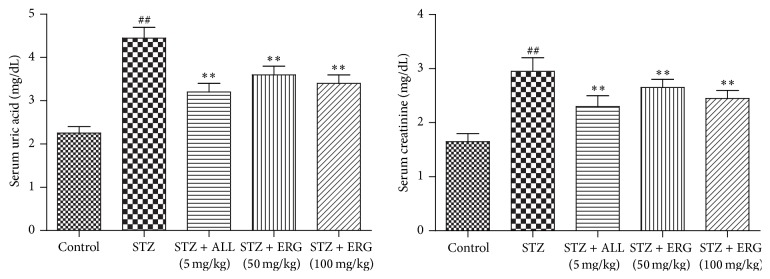
Effects of ERG on uric acid and creatinine concentrations. Mice were intraperitoneally injected with 135 mg/kg of STZ. ALL (5 mg/kg) and ERG (50, 100 mg/kg) were intragastrically treated for consecutive 28 days. Values are expressed as means ± SDs. Compared with control: ^##^ *P* < 0.01; compared with model: ^*∗∗*^ *P* < 0.01.

The concentrations of uric acid and creatinine in diabetes mice were relatively higher than those in the control group (*P* < 0.01). As expected, ERG treatment dramatically controlled the elevated levels of uric acid (*P* < 0.01) and creatinine (*P* < 0.01) in comparison with those in the model group (Figure 3). Little difference existed between ALL (5 mg/kg) and ERG (100 mg/kg) in the serum uric acid levels, while the level in ALL (5 mg/kg) group was less than that of ERG (50 mg/kg) group. Moreover, the serum creatinine level of ALL-treated group displayed slight declines compared to those of two ERG-treated groups.

### 3.4. Effect of ERG on Renal Pathological Changes

**Figure 4:**
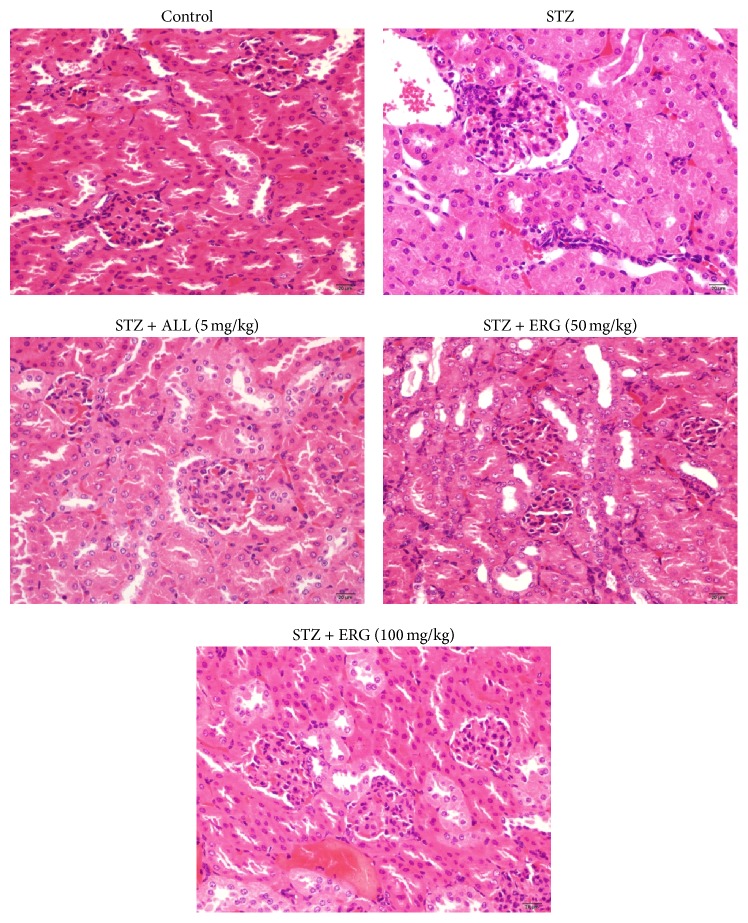
Effect of ERG on renal pathological changes. Mice were intraperitoneally injected with 135 mg/kg of STZ. ALL (5 mg/kg) and ERG (50, 100 mg/kg) were intragastrically treated for consecutive 28 days.

As exhibited in Figure 4, diabetes mice presented pathological changes as follows: thinned renal cortical, increased matrix in mesangium, vacuolar degeneration of glomerular epithelial cells, narrowed glomerular, thickened glomerular basement membrane, and capillary wall. Nevertheless, mice treated with ERG showed great improvement of the described symptom. Additionally, ALL treatment exerted more beneficial effect on renal tissues in comparison with those of ERG treatments.

### 3.5. Effect of ERG on PI3K/Akt/NF-*κ*B Pathway-Related Pathway

**Figure 5:**
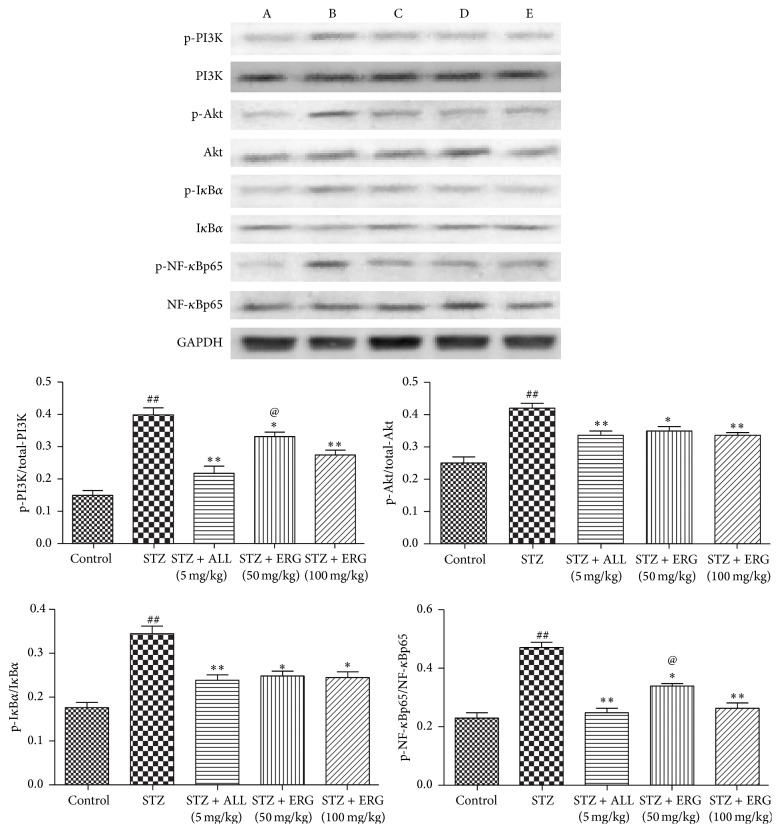
Effect of ERG on PI3K/Akt/NF-*κ*B pathway-related pathway. Mice were intraperitoneally injected with 135 mg/kg of STZ. ALL (5 mg/kg) and ERG (50, 100 mg/kg) were intragastrically treated for consecutive 28 days. Values are expressed as means ± SDs. Compared with control: ^##^ *P* < 0.01; compared with model: ^*∗*^ *P* < 0.05, ^*∗∗*^ *P* < 0.01. Compared with STZ + ERG (50 mg/kg) group: ^@^ *P* < 0.05. A: control; B: STZ; C: STZ + ALL (5 mg/kg); D: STZ + ERG (50 mg/kg); E: STZ + ERG (100 mg/kg).

To further investigate the possible antidiabetic mechanism, the expressions of PI3K/Akt/NF-*κ*B pathway-related proteins were also detected in renal. As presented in Figure 5, the expressions of p-PI3K, p-Akt, p-NF-*κ*Bp65, and p-I*κ*B*α* were upregulated in diabetes mice, taking the expressions of PI3K, Akt, NF-*κ*Bp65, and I*κ*B*α* as inner controls, respectively, (*P* < 0.01). On the contrary, these situations were obviously attenuated with the treatment of ERG and ALL (*P* < 0.01 or *P* < 0.1). Notably, when compared with the ERG-treated mice, the phosphorylated PI3K (*P* < 0.1) and NF-*κ*B (*P* < 0.1) were more effectively downregulated in ALL-treated mice compared with those in ERG (50 mg/kg) treated mice.

## 4. Discussion

Diabetes has long been a threat to human health and degrades the quality of life. An ideal antidiabetic drug should improve glucose metabolism and insulin resistance in diabetic patients without causing any adverse effect. Nevertheless, long-term therapy and prevention with conventional available antidiabetic drugs bring many negative side effects [17]. The World Health Organization Expert Committee on Diabetes has announced the great potential of natural plants and functional foods as alternative treatments for diabetes on the basis of over 400 reported traditional medicines [18]. Hence, the development of effective and safe natural resources against diabetes seems to be essential. Previous investigators found the antidiabetic effect of vitamin D [19] and the relationship between ergosterol and vitamin D [20]. As hypothesized, our findings demonstrated that ERG dramatically recovered the levels of blood glucose and insulin, as well as other biochemical indicators linked to diabetes symptoms in serum and urine.

The metabolic characteristics of diabetes include imbalanced glucose metabolism and insulin resistance, frequently accompanied by dyslipidemia [21]. The imbalance between glucose metabolism and insulin resistance in the current study could result from the damage stimulated by STZ, which works either directly or indirectly by enhancing the blood glucose level. Herein, the goals of managing diabetes mellitus are to balance the diet, optimize the control of blood glucose level, and normalize disturbances in lipid metabolism [22]. Consequently, the upregulated levels of glucose and downregulated levels of insulin were observed in diabetic mice in the present study, as suggested by the treatment of ALL which was usually applied as positive control medicine [23]. As expected, ERG significantly recovered blood glucose balance and elevated insulin concentration, which reflected the hypoglycemic effects of ERG on diabetic mice.

Hypercholesterolemia and hypertriglyceridemia in STZ-induced diabetic animals are well documented. Glucose in diabetic mice fails to convert to carbohydrates due to the lack of insulin. However, massive accumulation of glucose metabolizes to fatty acids in liver [24]. The overproduction of serum fatty acids by STZ-induced diabetics facilitates the conversion of excessive fatty acids into phospholipids and cholesterol in liver [22]. Accordingly, blood TC and TG levels are also strongly influenced. Since the normal levels of blood glucose and insulin levels in the blood were closely associated with blood TC and TG, the obtained data further verified the hypoglycemic and hypolipidemic effects of ERG on diabetic mice.

It is believed that diabetic patients present polydipsia, polyuria, and polyphagia. Prolonged osmotic diuresis may lead to excessive urinary electrolyte loss even renal dysfunction which is correlated with several abnormalities, including progressive renal failure and proteinuria [25]. As to our knowledge, previous studies documented that an elevated serum uric acid level independently predicts the development of chronic kidney disease such as diabetes because of glomerular hypertension and renal disease induced by the abnormal uric acid level [26]. In addition, Serum creatinine, as an important indicator of renal health, is produced by muscle metabolism which is excreted unchanged by the kidneys. Accordingly, in the study, elevated concentrations of uric acid and creatinine were found in urine of diabetic mice. In contrast, ERG treatment remarkably decreased the levels of uric acid and creatinine, which is consistent with renal pathological changes. Histological analysis presented that ERG (100 mg/kg) exhibited the more significant therapeutic effect of kidney on diabetes, with the inhibition of renal tubular epithelial cell swelling and glomerular enlargement. These renal data suggested that ERG might enhance kidney excretion to decline serum levels of uric acid and creatinine in diabetic mice. The findings were in accordance with previous literatures, which confirmed our hypothesis [27, 28].

PI3K/Akt pathway confers an important effect in the metabolic balance of insulin [29, 30]. The cellular events in muscle of diabetic mice lead to decreased glucose uptake and insulin resistance [31]. Upon binding to insulin resistance substrate proteins, PI3K, whose activity is essential for the insulin-stimulated increase in glucose transport, is activated [32]. NF-*κ*B is a significant node downstream of the PI3K/Akt pathway [33]. There is a notion that diabetes is a chronic systemic inflammatory disease and the induced oxidative stress exacerbated inflammatory response [34]. Besides, previous documents have revealed that Akt promotes IKK*α*/*β* phosphorylation in a mutual activation way and IKK*α*/*β* is essential for PI3K/Akt-mediated degradation of I*κ*B*α* [35]. Furthermore, it is reported that Akt possibly triggers the transactivation of NF-*κ*B through IKK*α*/*β* [36]. Thus, it is assumed that PI3K/Akt/NF-*κ*B pathway may be implicated in the development of diabetes and related complications.

Taking the relation between efficacy and dose into consideration, ALL was demonstrated to have a more inhibitory effect against diabetes in contrast to ERG. However, it also suggested the potential role of ERG in improvement of diabetes in the study. Taken together, ERG improves insulin level and relieves chronic inflammation through PI3K/Akt/NF-*κ*B pathway, thus decreasing the concentration of blood glucose. We revealed the potential that ERG might be an effective alternative treatment for diabetes and investigated the underlying mechanism of ERG in alleviating symptoms of diabetes in this study. Moreover, sufficient further research on ERG could be carried out.
